# C-reactive protein to lymphocyte ratio is a prognostic factor for unfavorable outcomes following aneurysmal subarachnoid hemorrhage

**DOI:** 10.1016/j.clinsp.2025.100778

**Published:** 2025-09-20

**Authors:** Yijun Lin, Sijia Li, Xingquan Zhao

**Affiliations:** a Department of Neurology, Beijing Tiantan Hospital, Capital Medical University, Beijing, China; b China National Clinical Research Center for Neurological Diseases, Beijing, China; c Research Unit of Artificial Intelligence in Cerebrovascular Disease, Chinese Academy of Medical Sciences, Beijing, China

**Keywords:** CLR, aSAH, Clinical outcomes, Hemorrhage, Stroke

## Abstract

**Purpose:**

The present study aimed to use the C-reactive protein-to-Lymphocyte Ratio (CLR) to investigate the impact of inflammation-immune imbalance on the prognosis of aneurysmal Subarachnoid Hemorrhage (aSAH) patients.

**Patients and methods:**

A total of 650 eligible aSAH patients were prospectively enrolled in this study. CLR was examined within 24 h after symptom onset and calculated by C-reactive protein levels (mg/L) divided by lymphocyte count (10^9^/L). The main outcome was 3-month unfavorable functional outcomes (modified Rankin Scale score of 4‒6). The secondary outcome was all-cause death (modified Rankin Scale score of 6). Multivariable logistic-regression analysis was performed to explore the relationship between CLR and clinical outcomes.

**Results:**

Univariate analysis revealed that elevated CLR levels were associated with poor functional outcomes (*p* = 0.03) and all-cause death (*p* < 0.01). After adjusting for potential confounding indices, higher CLR levels (> 6.02 mg/10^9^) were significantly associated with increased risk of 3-month poor functional outcomes (adjusted Odds Ratios: 2.89 [95 % CI: 1.02‒8.17]), but not related to all-cause death (adjusted Odds Ratios: 1.46 [95 % CI: 0.56‒3.80]).

**Conclusions:**

CLR during the hyperacute phase after aSAH might not only be utilized as a reliable biomarker to predict clinical outcomes of aSAH but also provide promising insight into future research to develop tailored treatments targeting inflammation-immune imbalance.

## Highlights

• Prospective study of 650 aSAH patients examining CLR levels within 24 h.
• Exploring CLR as a biomarker for inflammation-immune imbalance in aSAH.
• Elevated CLR levels are significantly linked to 3-month poor outcomes in aSAH.
• CLR as a biomarker for predicting outcomes and guiding treatments in aSAH.

## Introduction

Aneurysmal Subarachnoid Hemorrhage (aSAH) is a life-threatening neurological disease, affecting 8.09 million people worldwide.^1^ The global in-hospital mortality rate for aSAH is approximately 19 %‒20 %, with nearly one-third of survivors facing severe disability,^2^^,^^3^ imposing a substantial burden on families and societies. With the rapid development of treatment techniques, there has been a decreasing trend in overall aSAH disability and death.^1^ However, there are still patients at increased risk and experienced severe conditions and poor prognosis, which drives us to urgently explore the underlying prognostic factors in order to direct tailored treatment.

There is increasing evidence suggesting that inflammation significantly contributes to early brain injury following aSAH, which in turn leads to poor functional outcomes.^4^ Various biochemical markers can be used to detect the course of the inflammatory response after aSAH. C-Reactive Protein (CRP) is an acute phase protein produced by the liver and released into the blood under inflammation response and tissue injury.^5^ Previous studies have revealed that elevated levels of CRP were independently associated with poor outcomes in patients with aSAH.^6^ At the same time, lymphocytes play a key role in the anti-inflammatory response and cellular immune response.^7^ Prior research has demonstrated that some aSAH patients suffered from immunosuppression characterized by lower levels of lymphocytes.^8^ The C-reactive protein-to-Lymphocyte Ratio (CLR) is a composite novel marker that reflects both the inflammation response and immune defense, and it can be easily applied in clinical settings. In recent years, studies have reported that CLR can be utilized as a prognostic factor for pneumonia, pancreatitis, fever, and cancer.^9^, ^10^, ^11^ However, very limited research has shed light on the correlation between CLR levels and prognosis in patients with aSAH. Elevated CLR levels within 48 h after symptom onset were found to be significantly associated with poor functional outcome at discharge following aSAH in only one small series of < 300 patients.^12^ However, it remains uncertain whether CLR levels in the hyperacute phase could predict long-term functional outcome after aSAH. Therefore, the aim of the present study is to assess the predictive capability of CLR levels within 24 h after aSAH for 3-month outcomes.

## Methods

### Study population

This study was a prospective, observational cohort study involving patients with aSAH from the department of neurology emergency in Beijing Tiantan Hospital between October 2020 and July 2023. The research adhered to the ethical principles outlined in the Helsinki Declaration and received approval from the Institutional Review Board of Beijing Tiantan Hospital (KY2023–190–02). Written informed consent was obtained from all participants or their legally authorized representatives. Inclusion criteria were as follows: 1) Age ≥ 18 years, 2) Diagnosis of spontaneous SAH confirmed by head CT scan, 3) Presence of intracranial aneurysm confirmed by CT Angiography (CTA) or Digital Subtraction Angiography (DSA) and 4) Within 24 h after symptom onset. Exclusion criteria included: 1) SAH caused by other factors such as trauma, cerebral arteriovenous malformations, intracranial tumors, or moyamoya disease, 2) Previous history of ischemic or hemorrhagic stroke, vascular anomalies, or malformations, 3) Acute kidney injury or chronic kidney disease, 4) Concomitant systemic complications, including malignancy, cirrhosis, infection, or immune dysfunction.

### Baseline information

Baseline information, including demographics (age and gender), medical history (hypertension, diabetes mellitus, coronary heart disease, smoking and drinking), blood pressure and heart rate was all collected by trained physicians on admission. Smoking was defined as current smoking or having smoked regularly within the past year, while alcohol consumption was defined as regular drinking (at least one drink per week) in the past year.^13^ Neurological status was also assessed upon arrival using the Hunt-Hess scale and the World Federation of Neurosurgical Societies (WFNS) grade. On the initial CT scan, which was performed within 24 h after symptom onset, the authors recorded the extent of subarachnoid blood by the modified Fisher grade. The location and the morphology of the ruptured intracranial aneurysms were categorized on CTA or DSA according to established criteria.^14^^,^^15^

Treatment modalities, including endovascular coiling, surgical clipping, or conservative treatment, were determined by endovascular experts and experienced neurosurgeons based on the current guidelines.^1^ Moreover, the preferences of the patients’ families were also taken into account.

### Laboratory examinations and definition of CLR

Blood samples were drawn from an antecubital vein immediately upon arrival and before any treatment was conducted. Laboratory examinations, including White Blood Cells (WBC), lymphocytes, neutrophils, Hemoglobin (Hb), Platelets (PLT), and C-Reactive Protein (CRP), were collected for all participants by routine laboratory assays. CLR was calculated by CRP levels (mg/L) divided by lymphocyte count (10^9^/L).^10^

### Outcomes

All the patients were followed up by telephone interviews at 3 months after aSAH onset. Functional outcomes were assessed utilizing the modified Rankin Scale score (mRS) by trained research personnel who were blinded to baseline information. The primary outcome was unfavorable functional outcomes, defined as an mRS score of 4‒6.^14^ The secondary outcome was all-cause death, defined as an mRS score of 6.

### Statistical analysis

All statistical analyses were conducted using SPSS Statistics for Windows, version 22.0 (IBM Corp, Armonk, NY, USA) and *R* software (https://www.r-project.org/, version 4.1.2). The distribution of the continuous variables was checked using the Shapiro-Wilk test. Continuous parameters were described as means ± SD or medians with interquartile range based on the distribution of the data, while categorical parameters were expressed as numbers (percentages). Patients in the present study were separated into two groups according to their 3 months functional outcomes (mRS score 0‒3 vs. mRS score 4‒6) or all-cause death (survival vs. death). Chi-Squared tests were applied to perform comparisons for categorical variables. Student *t*-tests or Mann-Whitney *U* test were used for comparisons depending on the distribution of the continuous variables. Potential confounders with p-value < 0.2 in univariate analysis were entered into multivariable logistic regression models to identify the independent predictive factors for clinical outcomes. In addition, subgroup analysis was conducted by sex to evaluate the potential effects of CLR and sex on clinical outcomes. A two-sided *p* < 0.05 was defined as statistically significant.

## Results

**Fig. 1:**
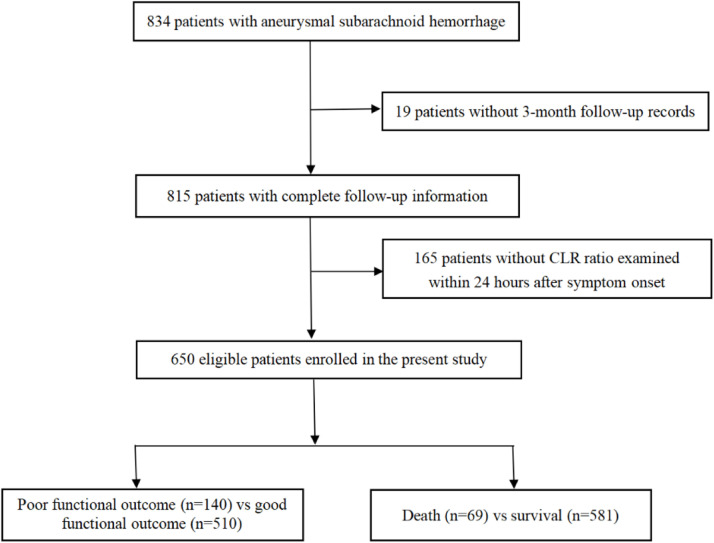
Flow chart of the study population. CLR, C-reactive protein to Lymphocyte Ratio.

**Fig. 2:**
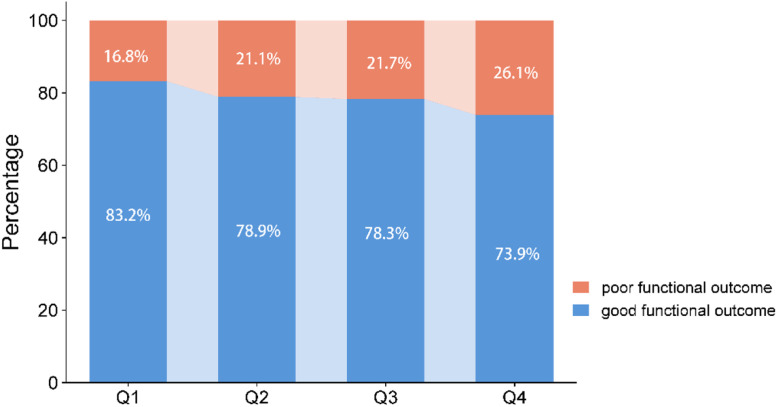
Incidence rates of good and poor functional outcomes in different CLR groups.

**Fig. 3:**
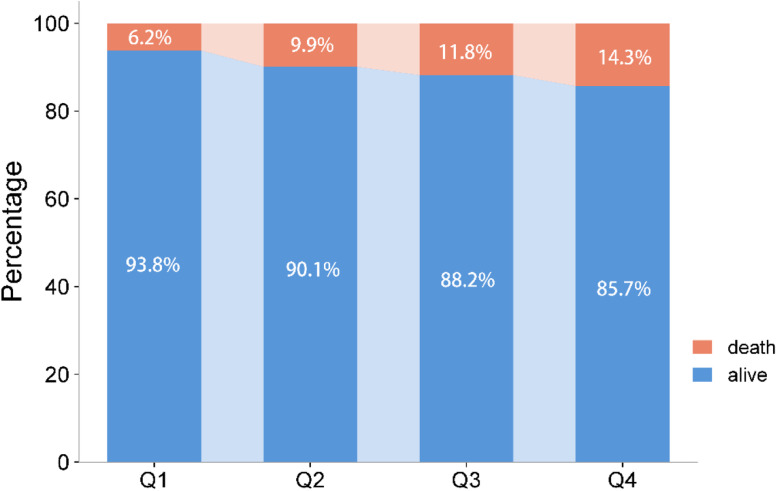
Proportions of mortality and survival rates among different CLR groups.

Among the 834 patients with aSAH, a total of 650 eligible patients with complete 3-month follow-up information and examination of CLR within 24 h after symptom onset were finally enrolled in this study (Fig. 1). The average age of these patients was 57.0 ± 12.4, of whom 420 were females (64.6 %). Worse functional outcome at 3-month was discovered in 21.5 % of the included patients, while 10.6 % of the patients died. The incidence rates of poor functional outcomes or all-cause death at 3-months in different CLR groups, stratified by quartile ranges (Q1: ≤ 0.96; Q2: 0.96‒2.38; Q3: 2.38‒6.02; Q4: > 6.02), are shown in Figs. 2 and 3. Patients from the higher quartiles of CLR were more likely to have an increased risk of 3-month poor functional outcome (Q1: 16.8 %, Q2: 21.1 %, Q3: 21.7 %, and Q4: 26.1 %) or all-cause death (Q1: 6.2 %, Q2: 9.9 %, Q3: 11.8 %, and Q4: 14.3 %).

Baseline characteristics grouped by 3 months poor functional outcome or all-cause death are presented in Table 1 and Supplementary Table 1, respectively. Patients with poor functional outcome were significantly older and had higher proportions of medical history such as hypertension, diabetes mellitus, coronary heart disease and smoking. Upon admission, elevated systolic blood pressure, faster heart rate, higher Hunt-Hess grade, WFNS grade, and modified Fisher grade, as well as higher proportions of multiple aneurysms were observed in the group with worse functional outcome. Furthermore, patients with unfavorable outcomes were more likely to have increased levels of WBC, neutrophils, CRP and CLR (Table 1). As shown in Supplementary Table 1, non-survivors were significantly older and were prone to have higher Hunt-Hess grade, WFNS grade, and modified Fisher grade upon admission. Additionally, laboratory indices such as WBC, neutrophils, CRP and CLR in the death group were much higher and non-survivors were inclined to receive conservative treatment.

**Table 1:** Baseline characteristics of the study population grouped by 3-month functional outcome.

	Total (*n* = 650)	Good functional outcome (*n* = 510)	Poor functional outcome (*n* = 140)	p
Age (years)	57.0 ± 12.4	54.7 ± 11.7	65.2 ± 11.3	<0.01
Female sex	420 (64.6)	327 (64.1)	93 (66.4)	0.61
**History**				
Hypertension	388 (59.7)	288 (56.6)	100 (71.4)	<0.01
Diabetes mellitus	70 (10.8)	46 (9.0)	24 (17.1)	<0.01
Coronary heart disease	56 (8.6)	32 (6.3)	24 (17.1)	<0.01
Current smoking	80 (12.3)	70 (13.7)	10 (7.1)	0.04
Alcohol	59 (9.1)	50 (9.8)	9 (6.4)	0.22
**Vital signs**				
SBP (mmHg)	154.0 (138.0‒169.0)	153.0 (137.3‒167.0)	159.0 (140.0‒176.8)	0.01
DBP (mmHg)	87.0 (79.0‒96.0)	87.0 (78.0‒97.0)	89.0 (80.0‒96.0)	0.19
Heart rate (/min)	79.0 (71.0‒88.0)	78.0 (70.0‒87.0)	83.0 (73.3‒91.0)	<0.01
**Neurological status**				
Hunt-Hess grade 3‒5	132 (20.3)	69 (13.5)	63 (45.0)	<0.01
WFNS grade 3‒5	74 (11.4)	30 (5.9)	44 (31.4)	<0.01
**Laboratory tests**				
WBC (× 10^9^/L)	12.3 (10.0‒15.1)	12.1 (9.8‒14.6)	13.4 (10.5‒16.8)	<0.01
Lymphocyte (× 10^9^/L)	1.0 (0.7‒1.3)	1.0 (0.7‒1.3)	1.0 (0.7‒1.2)	0.46
Neutrophil (× 10^9^/L)	10.9 (8.4‒13.5)	10.5 (8.3‒13.0)	12.2 (9.1‒15.2)	<0.01
Hb (g/L)	139.0 (128.8‒149.0)	139.0 (129.0‒149.3)	139.0 (126.3‒147.0)	0.29
PLT(× 10^9^/L)	231.0 (196.2‒270.0)	231.5 (196.0‒270.8)	231.0 (202.0‒270.8)	0.99
CRP (mg/L)	2.5 (0.9‒5.6)	2.4 (0.9‒5.6)	2.9 (1.1‒5.9)	0.04
CLR (mg/10^9^)	2.4 (1.0‒6.0)	2.2 (0.9‒5.8)	2.9 (1.1‒8.4)	0.03
**Aneurysm location**				0.33
Anterior cerebral artery	194 (30.0)	156 (30.6)	38 (27.1)	
Internal carotid artery	272 (41.8)	213 (41.8)	59 (25.2)	
Middle cerebral artery	117 (18.0)	94 (9.2)	23 (14.4)	
Posterior circulation	67 (10.3)	47 (9.2)	20 (14.3)	
**Aneurysm morphology**				0.35
Single-sac with smooth margin	166 (25.5)	138 (27.2)	28 (20.1)	
Single-sac with irregular margin	184 (28.3)	139 (27.4)	45 (32.4)	
Aneurysm with a daughter sac	163 (25.1)	128 (25.2)	35 (25.2)	
Multilobulated aneurysm	134 (20.6)	103 (20.3)	31 (22.3)	
Multiple aneurysm	137 (21.1)	92 (18.0)	45 (32.4)	<0.01
Modified Fisher grade 3‒4	347 (53.4)	239 (46.9)	108 (77.1)	<0.01
**Treatment**				<0.01
Coiling	270 (41.5)	239 (46.9)	31 (22.1)	
Clipping	297 (45.7)	239 (46.9)	58 (41.4)	
Conservative treatment	83 (12.8)	32 (6.3)	51 (36.4)	

**Notes:** Continuous variables are expressed as means ± (SD) or medians (IQR).

SBP, Systolic Blood Pressure; DBP, Diastolic Blood Pressure; WFNS, World Federation of Neurosurgical Societies; WBC, White Blood Cell; Hb, Hemoglobin; PLT, Platelet; CRP, C-Reaction Protein; CLR, C-reactive protein-to-Lymphocyte Ratio.

On multivariable analysis, CLR entered into the model as a categorical variable, stratified by four groups based on quartile ranges. Compared with patients in the lowest quartile of CLR, the odds ratio of the highest quartile (> 6.02 mg/10^9^) was 2.89 (1.02‒8.17) for the 3 months poor functional outcome after adjusting for age, sex, hypertension, diabetes mellitus, coronary heart disease, current smoking, blood pressure, heart rate, Hunt-Hess grade, WFNS grade, WBC, neutrophil, multiple intracranial aneurysms, modified Fisher grade and treatment methods (Table 2). However, elevated CLR levels (> 6.02 mg/10^9^) were not independently correlated with 3-month all-cause death (OR = 1.46; 95 % CI: 0.56‒3.80) after adjusting for age, sex, hypertension, current smoking, heart rate, Hunt-Hess grade, WFNS grade, WBC, neutrophil, aneurysm location, multiple intracranial aneurysms, modified Fisher grade and treatment method (Table 3).

**Table 2:** Crude and adjusted OR of CLR levels for 3-month poor functional outcome.

	Q1 (CLR ≤ 0.96)	Q2 (0.96 < CLR ≤ 2.38)	Q3 (2.38 < CLR ≤ 6.02)	Q4 (CLR > 6.02)
**Events, n (%)**	16.8	21.1	21.7	26.1
**Crude OR (95% CI)**	1.00 (reference)	1.33 (0.76‒2.33)	1.38 (0.79‒2.41)	1.75 (1.02‒3.01)
**Adjusted^a^ OR (95% CI)**	1.00 (reference)	2.53 (0.93‒6.86)	2.42 (0.84‒6.94)	2.89 (1.02‒8.17)

**Notes:**^a^Adjusted for age, sex, hypertension, diabetes mellitus, coronary heart disease, current smoking, blood pressure, heart rate, Hunt-Hess grade, WFNS grade, WBC, neutrophil, multiple intracranial aneurysms, modified Fisher grade and treatment methods.

.CLR, C-reactive protein-to-Lymphocyte Ratio; OR, Odd Ratios; CI, Confidence Interval.

**Table 3:** Crude and adjusted OR of CLR levels for 3-month all-cause death.

	Q1 (CLR ≤ 0.96)	Q2 (0.96 < CLR ≤ 2.38)	Q3 (2.38 < CLR ≤ 6.02)	Q4 (CLR > 6.02)
**Events, n (%)**	6.20	9.90	11.80	14.30
**Crude OR (95% CI)**	1.00 (reference)	1.67 (0.73‒3.79)	2.02(0.91‒4.49)	2.52 (1.16‒5.48)
**Adjusted^a^ OR (95% CI)**	1.00 (reference)	1.22 (0.45‒3.28)	1.74 (0.65‒4.67)	1.46 (0.56‒3.80)

**Notes:**^a^Adjusted for age, sex, hypertension, current smoking, heart rate, Hunt-Hess grade, WFNS grade, WBC, neutrophil, aneurysm location, multiple intracranial aneurysms, modified Fisher grade and treatment method.

CLR, C-reactive protein-to-Lymphocyte Ratio; OR, Odd Ratios; CI, Confidence Interval.

**Fig. 4:**
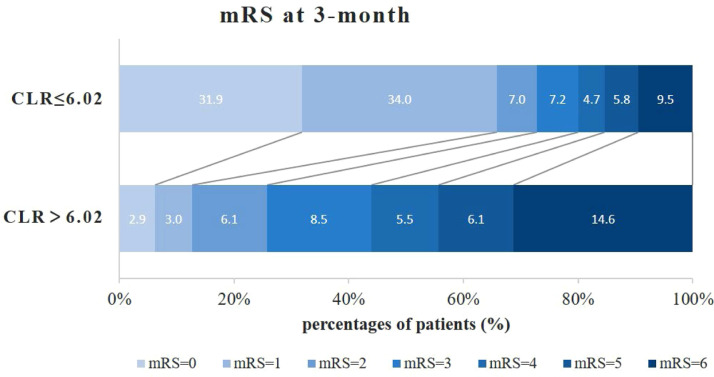
Distribution of mRS scores at 3-months stratified by CLR thresholds. mRS, modified Rankin Scale; CLR, C-reactive protein to Lymphocyte Ratio.

According to the threshold value used to independently predict 3 months poor functional outcome after aSAH, patients with CLR > 6.02 mg/10^9^ exhibited an increased proportion (26.2 %) of unfavorable clinical outcomes at the 3 months follow-up compared with patients with CLR ≤ 6.02 mg/10^9^ (19.96 %). The detailed distributions of the mRS score are shown in Fig. 4.

Subgroup analysis stratified by sex is shown in Table 4. The results showed that sex had no interaction effect on the correlation between CLR and poor clinical outcomes in patients with aSAH (all p-values for interaction > 0.05).

**Table 4:** Multivariate-adjusted OR and 95 % CI for poor clinical outcomes according to quartiles of CLR levels, stratified by sex.

Outcome	Subgroup	CLR				p for interaction
		Q1 (≤ 0.96)	Q2 (0.96‒2.38)	Q3 (2.38‒6.02)	Q4 (> 6.02)	
Poor functional outcome	Male	1.00 (reference)	1.61 (0.45‒5.84)	1.17 (0.28‒4.87)	3.03 (0.82‒11.19)	0.39
	Female	1.00 (reference)	1.24 (0.51‒3.02)	1.02 (0.43‒2.41)	0.87 (0.36‒2.12)	
All-cause death	Male	1.00 (reference)	1.45 (0.27‒7.01)	0.67 (0.10‒4.88)	3.51 (0.69‒17.95)	0.67
	Female	1.00 (reference)	1.81 (0.467‒7.07)	2.42 (0.65‒9.06)	1.67 (0.42‒6.71)	

OR, Odds Ratio; CI, Confidence Interval; CLR, C-reactive protein-to-Lymphocyte Ratio.

## Discussion

In this prospective cohort study of patients with aSAH, elevated CLR levels within 24 h after symptom onset were independently associated with increased risk of 3 months poor functional outcome, whereas no significant relationship was discovered between higher levels of CLR and 3-month all-cause death. The present findings suggest that CLR levels examined during the hyperacute phase after aSAH can be a reliable and novel biomarker to predict long-term functional outcome.

Many previous studies on the CLR levels mainly focused on the outcome of various cancers and infectious diseases.^11^^,^^16^^,^^17^ Prior research revealed that the CLR level was a feasible determinant of the overall survival in patients with gastric cancer, pancreatic cancer, and non-small cell lung cancer.^11^^,^^12^^,^^16^^,^^18^ Another study confirmed that CLR was a brand-new indicator for mortality outcomes in patients with severe fever with thrombocytopenia syndrome.^10^ However, only one study has been carried out to explore the association between CLR levels and prognosis after aSAH to date.^12^

In this retrospective analysis of 221 aSAH patients, CLR levels within 48 h of bleeding could independently predict unfavorable functional outcome at discharge. Due to the limitations of retrospective study design, small sample size, relatively longer time to blood sampling examination since symptom onset, and short-term outcome evaluation in this prior research, this study has extended the findings of this previous study. In the prospective and large cohort, the authors discovered for the first time that CLR levels examined within 24 h after symptom onset were significantly correlated with a 3-month poor functional outcome.

The potential explanations underlying the association of elevated CLR levels with increased risk of poor functional outcome after aSAH are as follows. First, an increase of CLR levels manifests as two parts: hyperactivation of the inflammation response, indicated by CRP elevations and immune disorders, indicated by decreased lymphocyte counts.^10^^,^^19^ On the one hand, the presence of blood in the subarachnoid cavity after an aneurysm rupture will activate a complex series of inflammatory molecules, contributing to the neuroinflammation cascade.^20^^,^^21^ CRP is an early-response protein produced by hepatocytes, which is increased in response to inflammatory cytokines, for instance, interleukin-6 and interleukin-1.^10^^,^^19^ Increased levels of CRP can also, in turn, augment the release of inflammatory cytokines, aggravating the vicious circle of the inflammation response.^17^ The hyperactivation of the inflammation response will further exacerbate brain injury, which ultimately results in neurological deficits.^20^ On the other hand, a large number of lymphocytes will be consumed after aSAH, which leads to a reduction in the total count of lymphocytes.^22^ Considering that lymphocytes play a key role in maintaining the homeostasis of the immune system,^10^^,^^23^ a growing body of evidence demonstrates that a temporary immunosuppression state is a crucial risk factor for infection complications after aSAH,^24^^,^^25^ contributing to the poor functional outcomes. In addition, the degree of lymphocytopenia is considered to be a sign of the severity of early brain injury.^26^ Thus, the authors speculate that decreased levels of lymphocytes may be associated with unfavorable functional outcomes.

As a convenient and readily available serum biomarker to predict poor functional outcome, CLR examined in the super-early phase of aSAH can help physicians to identify patients at increased risk early and activate prompt treatment to prevent further deterioration. For patients with elevated CLR levels, closer monitoring of neurological status and infectious complications, more rigorous intensive care treatment and more active follow-up after discharge should be performed to ameliorate prognosis in patients with aSAH to the maximum extent. Another clinical implication of the present findings is that elevated CLR levels indicate a severe imbalance between the inflammation response and the immune defense function; therefore, treatment of inflammation-immune disorders by targeting CRP or lymphocytes may have the potential to improve the prognosis of aSAH patients. A previous study has found that Dexmedetomidine (DEX) administration reduced IL-6 and CRP levels after SAH and ultimately attenuated neurological functional deficts.^27^ Furthermore, fingolimod (FTY720) has been reported to play a key role in retaining CD4+/CD8+ *T*-cells and central memory T-cells, which help to defend against infections.^28^ Although treatment targeting inflammation-immune imbalance has shown a promising future in several preclinical studies, the safety and efficacy of these therapeutic strategies still require more robust evidence and further exploration.

The present study also provides insights into the negative relationship between higher CLR levels and 3-month all-cause death after aSAH. A possible explanation might be that it is due to the predominant impact of clinical and radiological grading scales during the hyperacute phase on all-cause death. As reported before, the WFNS grade on day 1 was the second most important factor for predicting 1-year death after aSAH.^29^ Previous studies also suggested that the Hijdra sum score at the first CT scan, which reflected the amount of hemorrhage, demonstrated the highest diagnostic accuracy and robust predictive value for death during hospitalization.^30^ Therefore, the authors speculate that during the hyperacute phase, the traditional clinical and radiological grading system may weaken the predictive value of CLR levels in all-cause death. Further research is still needed to validate the association between CLR levels and long-term overall mortality after aSAH.

Previous studies have indicated that the incidence of aSAH is higher in females than in males,^31^^,^^32^ while the impact of sex on CLR and clinical outcomes in patients with aSAH remains unknown. In the present study, the authors found that there were no sex-specific differences in the relationship between CLR and poor prognosis after aSAH. This phenomenon can be explained by the fact that despite men demonstrating more severe inflammatory response and early brain injury after aSAH, possibly due to the lack of protective effects of estrogen, they also exhibit higher levels of anti-inflammatory gene expression compared to women.^33^, ^34^, ^35^, ^36^ That means that the augmented anti-inflammatory response in males counteracts the inflammation-induced brain damage to some extent, resulting in a comparable effect of CLR on clinical outcomes between males and females.

There are some limitations to be addressed in the present study. First of all, the cause-and-effect correlation between CLR levels and poor outcomes was not elucidated due to the observational nature of the study. Secondly, the dynamic changes of CLR levels were not examined, and the trajectory of CLR levels in relation to outcomes should be explored further. Finally, the single-center design of the present study may have introduced selection bias.

## Conclusions

In conclusion, the present study demonstrated that elevated CLR levels (> 6.02 mg/10^9^) were independently associated with unfavorable functional outcomes at 3 months in patients with aSAH. Moreover, CLR levels might not only be utilized as a convenient and easy-to-use serum biomarker to predict clinical outcomes of aSAH but also offer promising insight into future research investigating treatments that target the balance between inflammatory response and immune state.

## Ethics approval and informed consent

The study was approved by the Institutional Review Board of Beijing Tiantan Hospital, Captital Medical University (KY2023–190–02). All the participants or their legally authorized representatives signed the written informed consent. The study was conducted according to the ethical principles stated in the Helsinki Declaration.

## Consent for publication

Not applicable.

## Funding

The study was supported by Chinese Academy of Medical Sciences Innovation Fund for Medical Sciences (2019-I2M-5–029), 10.13039/501100001809National Natural Science Foundation of China (Grant no 82371302), and Capital Medical University Scientific Research Cultivating Funding (grant no PYZ23121).

## Data availability

The data that support the findings of this study are available from the corresponding author upon reasonable request.

## CRediT authorship contribution statement

**Yijun Lin:** Methodology, Formal analysis, Writing – original draft. **Sijia Li:** Conceptualization, Investigation, Writing – review & editing. **Xingquan Zhao:** Data curation, Validation, Supervision, Writing – review & editing.

## Declaration of competing interest

The authors declare no conflicts of interest.

## Appendix Supplementary materials

Image, application 1
