# Supplementary material for: C-reactive protein to lymphocyte ratio is a prognostic factor for unfavorable outcomes following aneurysmal subarachnoid hemorrhage
Source: Clinics (Sao Paulo). 2025 Sep 20;80:100778. doi: 10.1016/j.clinsp.2025.100778 (PMC12489826; doi:10.1016/j.clinsp.2025.100778)
Supplement: Supplementary file 1 [file mmc1.docx]

**CLINICS-D-24-00978_Supplementary Material**

**Supplementary Table 1** Baseline characteristics of the study population grouped by 3-month death.

|  | **Total**  **(n = 650)** | **Alive**  **(n = 581)** | **Death**  **(n = 69)** | **p** |
| --- | --- | --- | --- | --- |
| **Age (years)** | 57.0 ± 12.4 | 55.7 ± 11.8 | 67.5 ± 12.1 | <0.01 |
| **Female sex** | 420 (64.6) | 377 (64.9) | 43 (62.3) | 0.67 |
| **History** |  |  |  |  |
| Hypertension | 388 (59.7) | 340 (58.5) | 48 (69.6) | 0.08 |
| Diabetes mellitus | 70 (10.8) | 61 (10.5) | 9 (13.0) | 0.52 |
| Coronary heart disease | 56 (8.6) | 46 (7.9) | 10 (4.5) | 0.66 |
| **Current smoking** | 80 (12.3) | 75 (12.9) | 5 (7.2) | 0.18 |
| **Alcohol** | 59 (9.1) | 55 (9.5) | 4 (5.8) | 0.31 |
| **Vital signs** |  |  |  |  |
| SBP (mmHg) | 154.0 (138.0‒169.0) | 153 (137.8‒167.0) | 157.5 (140.3‒180.0) | 0.21 |
| DBP (mmHg) | 87.0 (79.0‒96.0) | 87.0 (78.0‒96.3) | 87.0 (80.0‒96.0) | 0.74 |
| Heart rate (/min) | 79.0 (71.0‒88.0) | 79.0 (70.5‒88.0) | 82.0 (73.0‒93.0) | 0.02 |
| **Neurological status** |  |  |  |  |
| Hunt-Hess grade 3‒5 | 132 (20.3) | 97 (16.7) | 35 (50.7) | <0.01 |
| WFNS grade 3‒5 | 74 (11.4) | 51 (8.8) | 23 (33.3) | <0.01 |
| **Laboratory tests** |  |  |  |  |
| WBC (×109/L) | 12.3 (10.0‒15.1) | 12.1 (9.9‒14.8) | 14.3 (11.3‒17.9) | <0.01 |
| Lymphocyte (×109/L) | 1.0 (0.7‒1.3) | 1.0 (0.7‒1.3) | 1.0 (0.7‒1.2) | 0.68 |
| Neutrophil (×109/L) | 10.9 (8.4‒13.5) | 10.5 (8.4‒13.2) | 12.7 (9.7‒16.1) | <0.01 |
| Hb (g/L) | 139.0 (128.8‒149.0) | 139.0 (129.0‒159.0) | 141.0 (127.0‒151.5) | 0.67 |
| PLT (×109/L) | 231.0 (196.2‒270.0) | 232.0 (197.5‒272.0) | 226.0 (189.0‒269.0) | 0.43 |
| CRP (mg/L) | 2.5 (0.9‒5.6) | 2.4 (0.9‒5.6) | 3.9 (1.7‒6.5) | 0.01 |
| CLR (mg/10^9^) | 2.4 (1.0‒6.0) | 2.3 (0.9‒5.8) | 4.0 (1.7‒9.0) | <0.01 |
| **Aneurysm location** |  |  |  | 0.05 |
| Anterior cerebral artery | 194 (30.0) | 180 (31.0) | 14 (20.3) |  |
| Internal carotid artery | 272 (41.8) | 243 (41.8) | 29 (10.7) |  |
| Middle cerebral artery | 117 (18.0) | 104 (17.9) | 13 (18.8) |  |
| Posterior circulation | 67 (10.3) | 54 (9.3) | 13 (18.8) |  |
| **Aneurysm morphology** |  |  |  | 0.98 |
| Single-sac with smooth margin | 166 (25.5) | 148 (25.6) | 18 (26.5) |  |
| Single-sac with irregular margin | 184 (28.3) | 166 (28.7) | 18 (26.5) |  |
| Aneurysm with a daughter sac | 163 (25.1) | 145 (25.0) | 18 (26.5) |  |
| Multilobulated aneurysm | 134 (20.6) | 120 (20.7) | 14 (20.6) |  |
| **Multiple aneurysm** | 137 (21.1) | 116 (20.0) | 21 (30.4) | 0.05 |
| **Modified Fisher grade 3‒4** | 347 (53.4) | 290 (49.9) | 57 (82.6) | <0.01 |
| **Treatment** |  |  |  | <0.01 |
| Coiling | 270 (41.5) | 260 (44.8) | 10 (14.5) |  |
| Clipping | 297 (45.7) | 279 (48.0) | 18 (26.1) |  |
| Conservative treatment | 83 (12.8) | 42 (7.2) | 41 (59.4) |  |

Continuous variables are expressed as means ± (SD) or medians (IQR).

SBP, Systolic Blood Pressure; DBP, Diastolic Blood Pressure; WFNS, World Federation of Neurosurgical Societies; WBC, White Blood Cell; Hb, Hemoglobin; PLT, Platelet; CRP, C-Reaction Protein; CLR, C-Reactive Protein-to-Lymphocyte Ratio.

**Supplementary Table 2** Multivariable logistic regression results for 3-month unfavorable functional outcome.

| **Variable** | **Adjusted OR (95% CI)** | **p-value** |
| --- | --- | --- |
| Age | 1.07 (1.05‒1.10) | <0.01 |
| Sex | 0.74 (0.43‒1.27) | 0.28 |
| Hypertension | 1.14 (0.68‒1.90) | 0.63 |
| Diabetes mellitus | 1.40 (0.72‒2.74) | 0.32 |
| Coronary heart disease | 1.50 (0.71‒3.15) | 0.28 |
| Current smoking | 0.42 (0.16‒1.08) | 0.07 |
| Heart rate | 1.02 (1.00‒1.04) | 0.02 |
| SBP | 0.99 (0.98‒1.01) | 0.67 |
| DBP | 1.02 (0.99‒1.04) | 0.06 |
| WBC | 1.02 (0.87‒1.19) | 0.85 |
| Neutrophil | 1.05 (0.90‒1.22) | 0.52 |
| Multiple intracranial aneurysms | 1.68 (0.99‒2.83) | 0.05 |
| Modified Fisher grade | 1.51 (1.18‒1.95) | <0.01 |
| WFNS grade | 1.63 (1.20‒2.19) | <0.01 |
| Hunt-Hess grade | 1.13 (0.74‒1.72) | 0.57 |
| Treatment methods | 0.94 (0.66‒1.33) | 0.71 |
| CLR | 1.01 (1.00‒1.02) | 0.05 |

SBP, Systolic Blood Pressure; DBP, Diastolic Blood Pressure; WFNS, World Federation of Neurosurgical Societies; WBC, White Blood Cell; CRP, C-Reaction Protein; CLR, C-Reactive Protein-to-Lymphocyte Ratio.

**Supplementary Table 3** Multivariable logistic regression results for 3-month all-cause death.

| **Variable** | **Adjusted OR (95% CI)** | **p-value** |
| --- | --- | --- |
| Age | 1.07 (1.04‒1.10) | <0.01 |
| Sex | 0.55 (0.27‒1.22) | 0.1 |
| Hypertension | 0.90 (0.45‒1.81) | 0.77 |
| Diabetes mellitus | 0.57 (0.22‒1.51) | 0.26 |
| Coronary heart disease | 0.67 (0.0.25‒1.86) | 0.45 |
| Current smoking | 0.43 (0.12‒1.58) | 0.2 |
| Heart rate | 1.01 (1.00‒1.03) | 0.38 |
| SBP | 1.01 (0.99‒1.02) | 0.39 |
| DBP | 1.01 (0.98‒1.04) | 0.56 |
| WBC | 1.08 (0.89‒1.29) | 0.44 |
| Neutrophil | 1.05 (0.88‒1.25) | 0.59 |
| Multiple intracranial aneurysms | 1.19 (0.59‒2.40) | 0.62 |
| Modified Fisher grade | 1.43 (0.99‒2.04) | 0.05 |
| WFNS grade | 1.15 (0.79‒1.69) | 0.46 |
| Hunt-Hess grade | 1.40 (0.78‒2.50) | 0.26 |
| Treatment methods | 0.38 (0.24‒0.61) | <0.01 |
| CLR | 1.02 (1.00‒1.03) | 0.03 |

SBP, Systolic Blood Pressure; DBP, Diastolic Blood Pressure; WFNS, World Federation of Neurosurgical Societies; WBC, White Blood Cell; CRP, C-Reaction Protein; CLR, C-Reactive protein-to-Lymphocyte Ratio.
